# Supplementary figures and images for: Widespread emergence of OmpK36 loop 3 insertions among multidrug-resistant clones of Klebsiella pneumoniae
Source: PLoS Pathog. 2022 Jul 11;18(7):e1010334. doi: 10.1371/journal.ppat.1010334 (PMC9302836; doi:10.1371/journal.ppat.1010334)

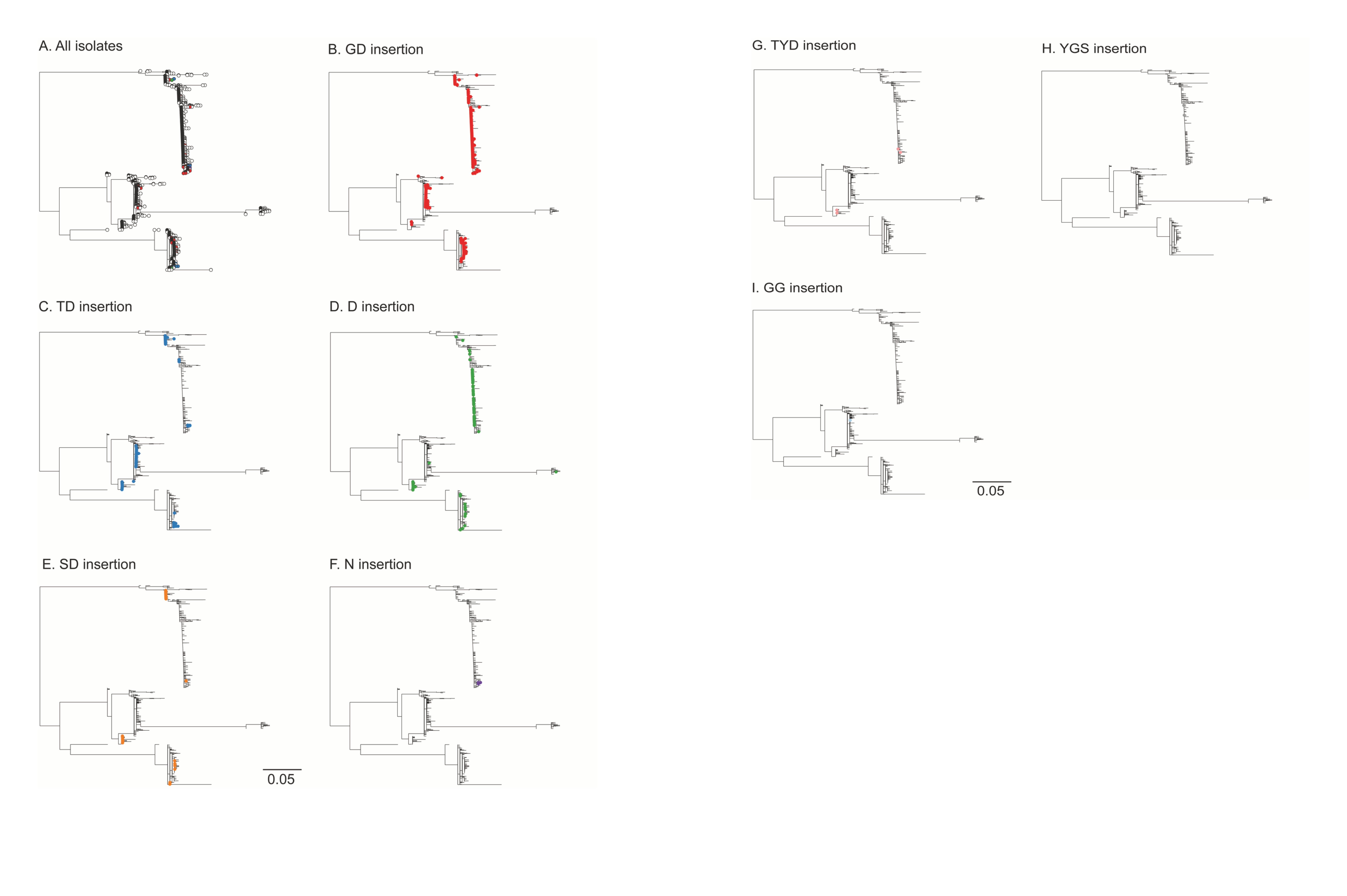

Supplement: S1 Fig — Midpoint-rooted phylogeny of intact ompK36 genes from 14,888 KP isolates with highlighted nodes of all gene variants (A) and only those harbouring a specific L3 insertion type (B-I). (TIFF) [file ppat.1010334.s001.tiff]

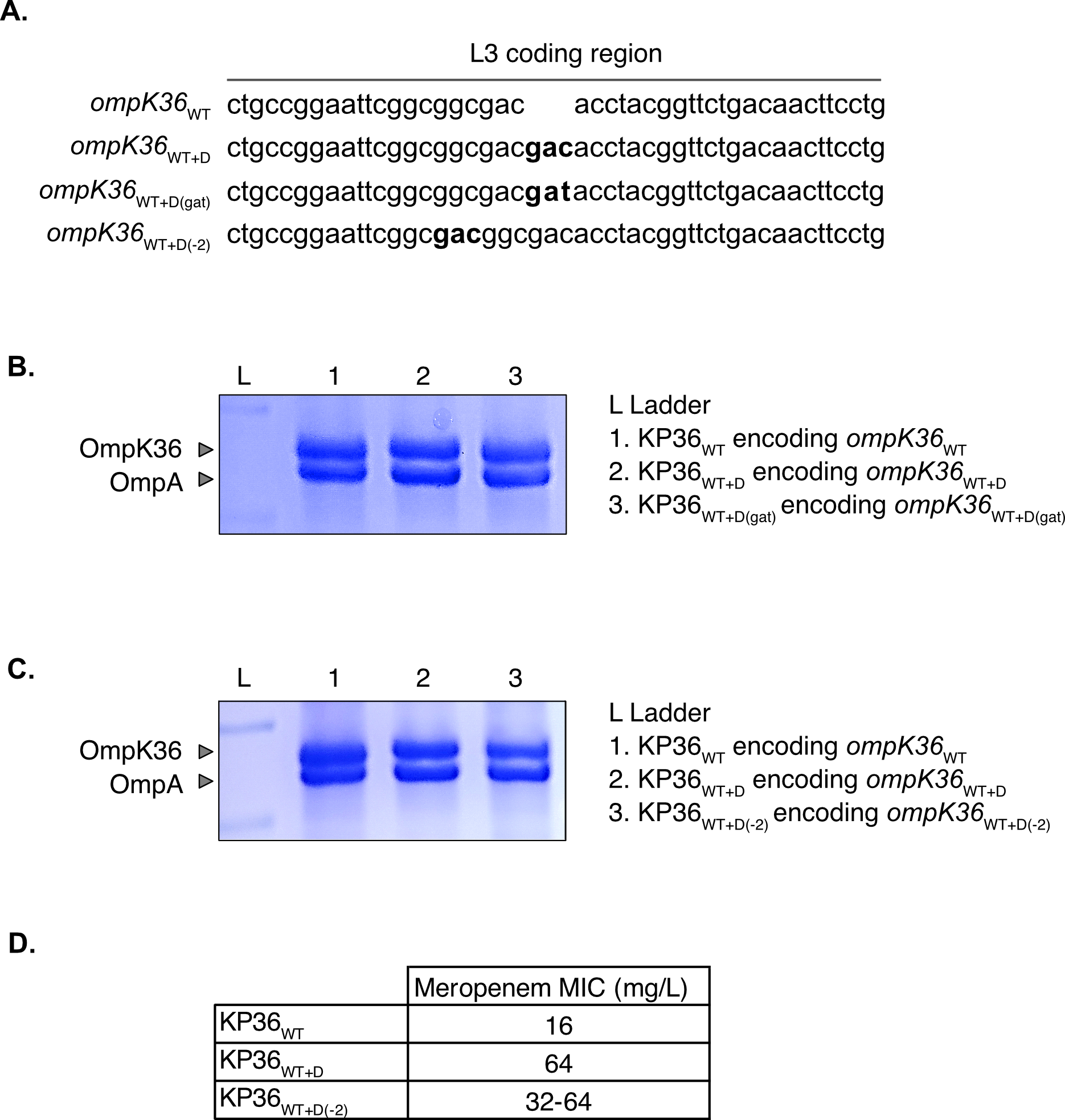

Supplement: S2 Fig — (A) The D insertion in OmpK36WT+D is mediated by an additional gac codon in the L3 coding region of OmpK36. We generated a codon switch mutant in which this additional gac was replaced with a synonymous gat codon and positional mutant in which the gac codon was moved -2 positions backwards, towards to 5’ end of the ompK36 open reading frame (towards the N-terminal). (B) Outer membrane preparations (OMP) separated by sodium dodecyl sulfate–polyacrylamide gel electrophoresis (SDS-PAGE) and Coomassie staining demonstrating no change in OM OmpK36 abundance in with OmpK36WT+D in which the insertion is encoded by a gac or gat codon). (C) OMP followed by SDS-PAGE separation and Coomassie staining demonstrate no change in abundance when the D insertion is moved in position which functionally results in a non-significant change in the meropenem MIC (D). All strains in D have ompK35 deleted and express KPC-2 from a pKpQIL-like plasmid. (TIFF) [file ppat.1010334.s002.tiff]

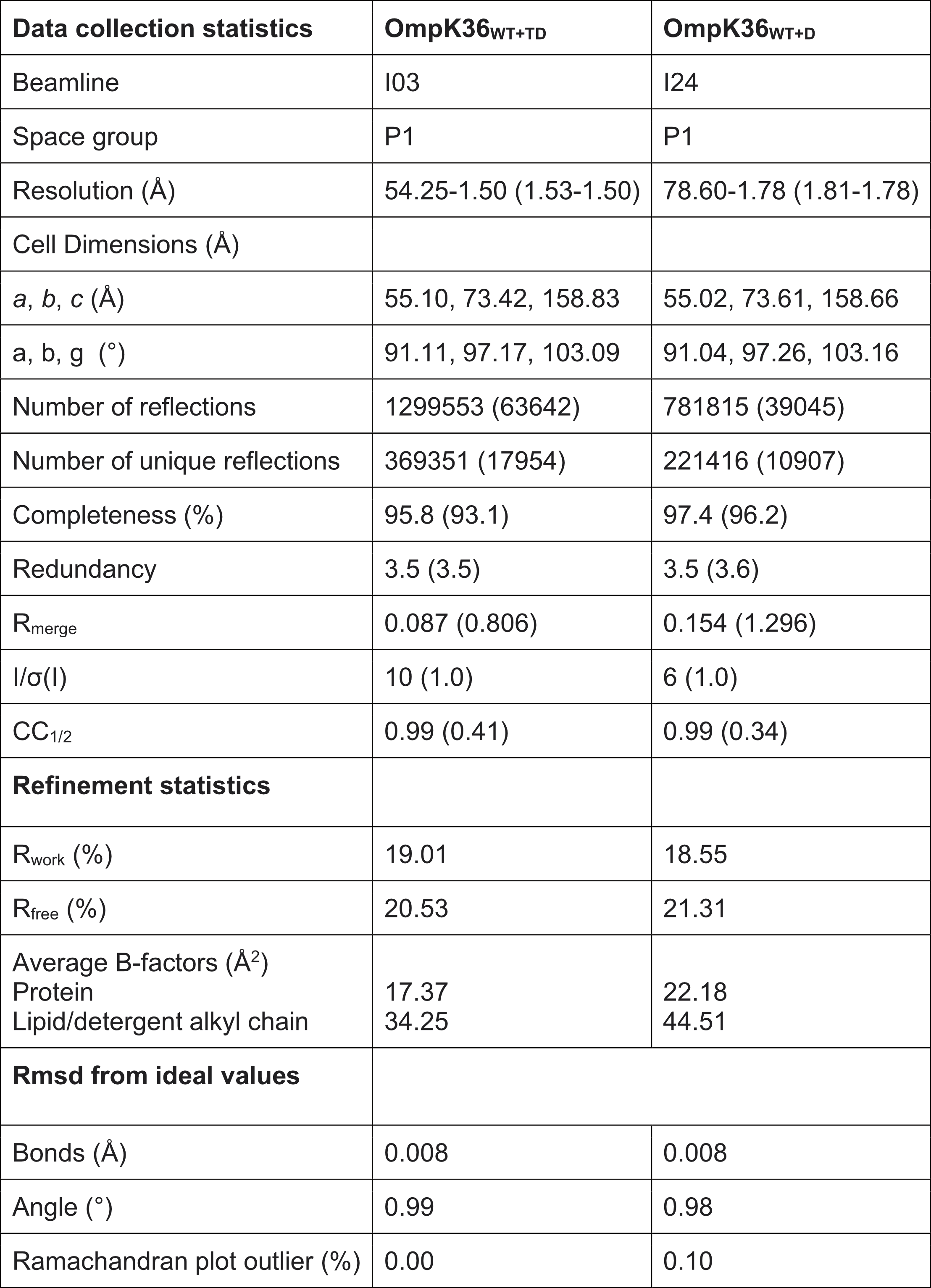

Supplement: S2 Table — Value in parenthesis refer to data in the highest resolution shell. (TIFF) [file ppat.1010334.s004.tiff]

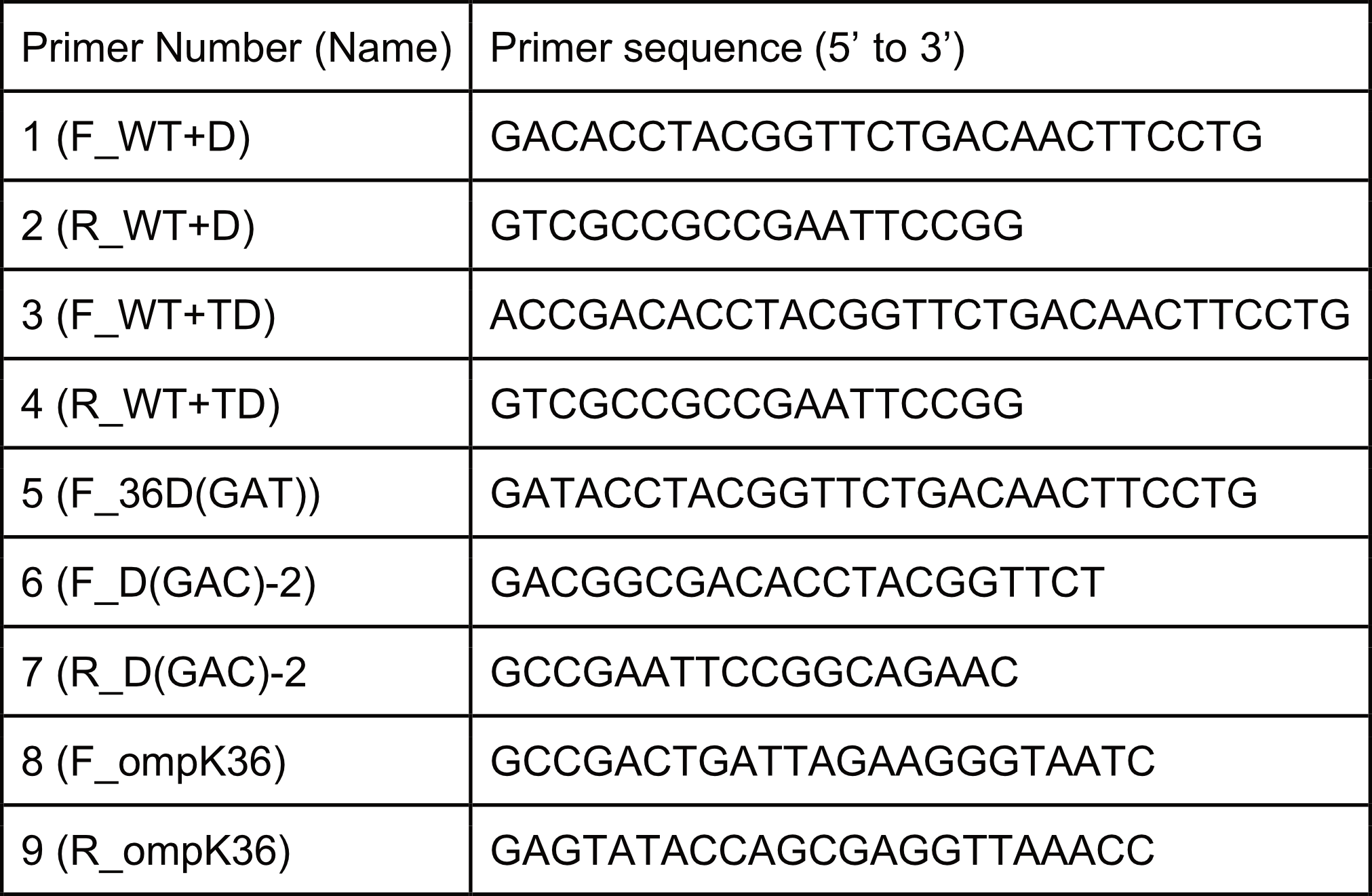

Supplement: S3 Table — (TIFF) [file ppat.1010334.s005.tiff]
