# Supplementary material for: Widespread emergence of OmpK36 loop 3 insertions among multidrug-resistant clones of Klebsiella pneumoniae
Source: PLoS Pathog. 2022 Jul 11;18(7):e1010334. doi: 10.1371/journal.ppat.1010334 (PMC9302836; doi:10.1371/journal.ppat.1010334)
Supplement: Attachment — Submitted filename: PlosPathogens_ReviewersComments_revision.pdf [file ppat.1010334.s006.pdf]

We would like to thank the editor and the reviewers for their constructive and thoughtful critique of the data. Our responses to individual comments are listed below; changes in the text are highlighted in yellow in the word document "Manuscript_revised_marked":

Response to comments made by the editors

Comment 1: An important concern raised in the reviews is about the novelty of the major findings where the results are to some extent confirmatory of the authors' previous work on the effect of GD insertions on porin structure as well as the fact that these insertions have been noted in other studies.

Response: Our previous work is now summarised to delineate what is novel and what builds on previous findings in the introduction (lines 65-78); lines following this summary (78-90) refer to what is known on porin structure up until this publication, and the limitations and gaps in the knowledge that are to be addressed. We hope this clarifies this point.

Comment 2: Nevertheless, the novelty of the findings would be strengthened by a more extensive analysis of nutrients besides glucose that could have differential permeability between wild-type compared to L3 insertion mutants of OmpK36 strains hereby giving a mechanistic basis for difference in fitness.

Response: We have now proceeded to test this *in vitro* and show for the first time the transport of amino acids and small peptides (cas-aminoacids) are transported via OmpK36 and that an L3 insertion mediated competitive disadvantage (lines 262-275, new Figure 4 G-I).

Comment 3: Ideally, a gut colonization experiment would address any concerns regarding transmission and would strengthen the impact of their findings. The authors need to address the concern regarding apparent conflicting statements regarding fitness of L3 insertion mutants.

Response: We have modified the language used to describe transmission and agree that a gut colonisation experiment would have been helpful in achieving this. We now refer to observations from KP gut colonisation models in the introduction (line 91-104) and have included a sentence (line 406-412) in the discussion as to why we did not pursue this. Indeed, there are no good small animal models of gut KP carriage, and all rely on antibiotic pressure throughout the colonisation window. In order to clarify this point and to aid the reader we have introduced the idea of context-dependent selection (where the context is antibiotics throughout). We hope this addresses the point and thank the reviewer for helping improving the clarity in the manuscript.

Response to main comments made by reviewer 1:

Comment 1: Authors suggest a defect is only observed during in vivo competition. The single mutants colonize as WT, whereas in coinfection the WT outcompetes the mutant, potentially suggesting that there is a biological cost associated with L3 mutations that become apparent when there is competition for nutrients. To provide insight into this, authors should carry out competitive growth experiments in minimal media with either glucose or another carbon source. With a smaller pore size for the mutant it is possible that even though glucose can enter, its entry kinetics are different than WT, which would provide molecular reason for reversion to occur when antibiotic pressure is removed.

Response: We thank the reviewer for this suggestion; in response we have performed *in vitro* competition assays with cas-amino acids and lactose. These showed that OmpK36 L3 insertions also impact on the diffusion of larger substrate molecules, including amino acids and carbohydrates, while not impacting the usage of glucose as the carbon source, giving KP expressing WT OmpK36 a competitive advantage. For visual clarity we did not add non-significance to graphs, but these results can now be seen in Figure 4G-I and are described in lines 262-275.

Response to major comments made by reviewer 2:

Comment 1: I found the discussion of fitness costs very confusing and apparently contradictory in places. Please see the quotes from different lines in the manuscript below (not included here):

I understand that there appears to be a genetic context-dependence of the fitness costs, and also that the fitness costs are more apparent in the murine competition experiment than in the single isolate infection experiments. But I think the findings need to be summarized in a way that appears consistent without blatant contradictions. If the conclusion is as in lines 188-191 that reversions are frequent and in lines 241-243 that there is a significant fitness disadvantage, then the Author Summary in Line 34 should not conclude "L3 insertions impose only a low fitness cost" and lines 169-170 should not state that L3 insertions…revert at a low frequency. I would suggest rewriting the sections pertaining to fitness costs so that they do not appear contradictory.

Response: We thank the reviewer for these important comments that we have now taken on board throughout the text, clarifying the sections on fitness costs (e.g. line 288, 431) and introducing the theme of context dependence (where the context is carbapenem antibiotics, in which context L3 insertions are beneficial; e.g. lines 209, 315-318).

Comment 2: I also believe the authors need to do a better job with defining exactly what the novel reportable findings and biology are in this work. The bioinformatics study is expansive and well-done, but the main results are not really new as the most common insertions have been noted in many other studies. The structural analysis of the T and TD insertions is well done, but may represent a somewhat incremental advance over the authors' prior work on the GD insertion and structural work on Omps done by other groups. There is also a more general literature on fitness costs of Omp mutations and deletions. I believe that adding some focusing sentences in each section that highlight the new findings (as opposed to those generally confirmatory of principles already understood) would be very helpful.

Response: We understand the reviewer's point of view and have now expanded the introduction (lines 65-78) to describe our and others' previous work. Whilst it appears a technologically straightforward incremental advance, the severe pore constriction in the TD insertion is novel and will guide rational drug design. Our aim was to provide an overarching picture which included a multidisciplinary assessment of OmpK36 mutations from genomics to molecular biology to *in vivo* modelling. We believe that by integrating this information into one piece this in itself represents a significant increase knowledge in the field. We also now add in additional data to further increase the impact of this project including:

1. Evidence of nitrogen source diffusion across OmpK36 in addition to carbohydrate sources (Figure 4G-I, line 262-275).
2. Evidence that L3 insertion (all) affect the transport of these nitrogen sources.

3. Evidence that L3 insertion nature (position and coding) is not related to codon optimisation or functional implications of insertions at a different site within L3. (Figure S2, lines 146-162)

Response to major comments made by reviewer 3:

Comment 1: Line 108 – It is stated based on the phylogenetic analysis in Figure S1 that the different L3 deletions emerged multiple times independently, suggesting that "these underlying mutations are more likely to evolve than alternatives". First, there is no evidence presented that they are more likely to evolve, but perhaps more prone to spread. Second, while Figure S1 indicates that convergence is unlikely due to homologous recombination of the entire ompK36 gene, it does not rule out recombination of regions containing the L3 deletions. I would suggest exploring this possibility, as has been done recently for toxin allele switching in C. difficile (Mansfield et al., PLos Pathogens, 2020).

Response: Indeed, these are important points. We now clarify our findings regarding the role of recombination and *de novo* mutation in the generation and spread of L3 insertions (note that we are discussing "L3 insertions" and not "L3 deletions"). We actually did find evidence that supports a role for homologous recombination in the sharing of L3 insertions, as a phylogenetic tree constructed using 14,888 intact *ompK36* sequences from our global collection shows some clustering of *ompK36* genes with particular L3 insertions from distantly related STs. We have re-worded the paragraph (lines 137-145) to make this finding more prominent and also added a Microreact link where the data can be explored and these particular findings observed.

We also agree with the reviewer that the parallel emergence of particular L3 insertions across different *ompK36* backgrounds, as observed in the phylogenetic analysis described above, could potentially be explained by intragenic recombination as well as by *de novo* mutation. However, the very distinct nature of many of the *ompK36* variants with a particular L3 insertion (i.e. large separation in the *ompK36* tree) suggests that any potential region(s) of intragenic recombination would need to be very small (i.e. make up only a small fraction of the *ompK36* sequence used to build the tree). Further to this, the L3 region and surrounding sequence have limited diversity and, as such, we have been unable to find any supporting evidence of intragenic recombination. To address the reviewer's concern, we have therefore adjusted our language in our description of the role of *de novo* mutation (i.e. [the data] "**may** suggest an additionally important role for *de novo* mutation"). For the sake of clarity and brevity, we would rather avoid discussing intragenic recombination here as this paragraph is not central to the paper narrative but merely seeks to point out an interesting observation that the underlying mutations of L3 insertions are always the same. However, we have also further explored the role of recombination in the acquisition and reversion of L3 insertions in STs 258/512, 16 and 231 and discuss the possibility of intragenic recombination there (see the response to Comment 5 below).

Finally, we agree that the homogeneity of the L3 insertions could also reflect an increased tendency of these variants to spread, as well as evolve, and have thus clarified this in the text.

Altogether the paragraph (lines 133-145)-now reads:

"L3 insertions were found in a total of 68 STs (GD - 52 STs, TD - 16 STs, D - 15 STs, SD - 10 STs, N - 1 ST, TYD - 3 STs, YGS - 2 STs), demonstrating their widespread emergence across the KP population. Notably, despite genetic redundancy, we found that the coding mutations for

each L3 insertion were always the same (e.g. GD always encoded by ggc gac, TD by acc gac and D by gac). This homogeneity in the coding mutations may be at least partially explained by recombination, as evident from phylogenetic analysis of the ompK36 open reading frame (ORF) that demonstrates some sharing of alleles between distantly related STs (https://microreact.org/project/52zjajyXDYr2ABfaaG2kaV-ompk36-gene-n14888). However, this analysis also showed parallel emergences of each insertion type across different ompK36 gene backgrounds (Figure S1). This may suggest an additionally important role for de novo mutation in the generation of L3 insertions; in addition, this implies that the particular underlying coding mutations of each L3 insertion type are more likely than the possible alternatives to evolve and/or spread."

Comment 2: On line 81 in the introduction, and repeatedly throughout the rest of the manuscript (e.g. line 200, line 204, etc.), it is stated that L3 deletions drove large clonal expansions of resistant clones of K. pneumoniae. However, there are several issues that prevent such a conclusion. First, as stated by the authors, the L3 deletions are linked to carbapenemase acquisition and also ompK35 deletions, making it impossible to isolate the specific effect of L3 deletions to lineage success. Arguing for the role of the carbapenemases themselves (or other genes/mutations co-acquired with them), there are several prominent lineages and sub-lineages that harbor those genes and have WT ompK36. A second issue with inferences regarding clonal expansions are severe observation biases within sequence databases for antibiotic resistant strains. In other words, it doesn't seem possible to isolate the impact that ompK36 deletions had on lineage success, because studies often bias themselves by phenotypically screening for isolates that have these mutations. Finally, public databases have other types of sampling biases (e.g. over sequencing of outbreaks in certain geographies), which aren't considered when making these claims.

Response: We thank the reviewer for raising these points. We agree that the effect of *ompK36* L3 insertions on lineage expansion cannot be isolated from the effects of carbapenemase acquisition and *ompK35* deletions, and also that genome collections likely overestimate the prevalence of L3 insertions due to targeting of sampling/sequencing towards antibiotic-resistant strains. However, analysis of the large, diverse collection of global genomes nevertheless shows a clear association of L3 insertions with major, clinically relevant STs which represent large clonal expansions across the *K. pneumoniae* species (e.g. Fig. 1C shows a clear concentration of L3 insertions in STs 258/512, ST16 and ST231). Thus we have altered statements to clarify that L3 insertions are associated with clonal expansions but removed reference to L3 insertions specifically having driven them (i.e. lines 109, 186, 200…).

Comment 3: To understand the overall stability of the ompK36 L3 deletions, along with their occasional convergent loss, the authors use a mouse pneumonia model. I have several concerns about this. First, the authors provide no justification for why this is a relevant model for providing insight into the transmission dynamics of Klebsiella mutants. Given that the presumed reservoir for transmission is the gut, it would seem a gut colonization model would be more appropriate. Second, the authors conclude that the lack of impact of L3 deletions on virulence explains why L3 deletions are stable. However, the rationale for this is unclear, as to my knowledge there is not a known association between severity of or propensity to cause respiratory infections, and epidemic success of K. pneumoniae lineages. Third, the authors state that the competitive advantage of WT versus L3 mutants in this respiratory model explains convergent reversion to WT in L3 mutant lineages. Again, there is no evidence that

respiratory colonization/infection is the underlying selective pressure in human isolates. This would perhaps be more convincing if isolates with L3 reversions were enriched in respiratory isolation. Overall, these mouse experiments feel a bit like just so storytelling, where the outcome of the experiments are being interpreted to fit a story. However, there are a myriad of alternative hypotheses to explain selective pressures underlying proliferation and loss of L3 deletions were not explored.

Response: We would like to note that we observe no L3 deletions. This would structurally result in a porin with very wide lumen which we believe would not be tolerated in the KP OM. For clarity, we have modified the text throughout and added thematically context-dependence (e.g. lines 209, 315-318). L3 insertions are positively selected as they result in increased carbapenem MICs; however, the competitive fitness disadvantage (in the absence of antibiotics in this model) may underpin why spontaneous reversions are observed in the phylogeny. We use the pneumonia model to do this as 1. It demonstrates there is a fitness cost associated with L3 insertions which is not apparent during single inoculum infections, and it is a more stringent test of fitness. 2. This is the site that results in morbidity and mortality with KP infections.

Unfortunately, there is no metadata uniformly collected across the collection to allow us to specifically analyse the impact of L3 insertions specifically in respiratory isolates.

We have also added into the text the limitations associated with gut colonisation models (lines 91-104) and why we chose not to utilise at a gastrointestinal colonisation model (lines 406-412). The conditioning required to establish 'colonisation' in mice is the exact context in which we propose such L3 insertions would have an advantage. No mouse KP strain exists which is able to colonise/integrate into the microbiome/commensalise in the absence of antibiotic clearance or disruption of the endogenous microbiome.

Comment 4: When evaluating the function of porin mutants, why was glucose uptake measured? Are there other relevant nutrients taken up through this channel that might help account for fitness costs?

Response: Glucose is used throughout the field as a positive control in liposomal swelling assays as it is small and hydrophilic. We chose to use this in line with all previous work and also because it is a carbon source available within the host.

In response to the reviewer's comment we have added new data of nitrogen source diffusion and L3 insertion mediated inhibition of this process (Figure 4G-I, line 262-275).

Comment 5: A bit more analysis on the genetic pathways for reversion of L3 deletions and their proliferation would be interesting. In particular, is there evidence that reversions are due to recombination mediated allele switching, and once these reversions occur, are they always dead ends?

Response: Again, it should be noted that the manuscript is describing L3 "insertions" and not "deletions".

We have now investigated the recombination events detected by Gubbins in the three major lineages that we describe in detail (ST258/512, ST16 and ST231) and investigated whether any events can be linked to both the reversion but also initial acquisition of L3 insertions.

To this end, we have now added the following text to the manuscript:

[lines 190-194] "We included genomes from the Pathogenwatch collection belonging to each of these STs, which represented 3629 ST258/512 isolates (34 countries; collected 2003-2020), 446 ST16 isolates (26 countries; 2004-2020) and 302 ST231 isolates (19 countries; 2003-2019), and constructed a phylogeny of each after the removal of recombined regions using Gubbins."

[lines 229-237] "Finally we investigated the role of recombination in the acquisition and reversion of L3 insertions in these three major lineages by assessing the recombined regions identified by Gubbins. While no recombination events involving *ompK36* were found in ST231, three were identified in ST258/512 and five in ST16. However, none of these events could be linked to either the acquisition or reversion of L3 insertions via a comparison of the affected clades. This would further imply a prominent role for *de novo* mutation, as previously suggested by the analysis of the global KP collection, although recombination involving closely-related strains or affecting only a small part of the gene (which Gubbins may be unable to detect) also cannot be ruled out."

Regarding whether reversions are dead ends, we do not think there is a reason to suspect this would be the case since we show that the majority of KP isolates indeed lack an L3 insertion and proliferate nonetheless. It is also evident from Figures 3 and 4 that, while reversions are often observed in singleton isolates, this is not always the case. For these reasons, we would rather avoid adding any text to the manuscript on this specific point.

Response to minor comments made by reviewer 1:

Comment 1: Were any statistical differences observed in Fig 2F between the WT and mutants. The statistics are only shown between empty liposome and variants. The GD variant does have a small defect, which would again explain their in vivo competition experiment.

Response: We did carry out multiple comparison and there is no significant difference between any of the OmpK36 variants (ordinary one-way ANOVA with Tukey's multiple comparison test). For clarity we have added this to the figure legend (now Figure 4F, line 930). We would prefer not to have all NS's added into the figure to aid the reader, as the number of comparison lines on the graph would interfere with understanding.

Comment 2: It is not clear from the manuscript, how the mutants were constructed. The reviewer assumes that the OmpK36 mutations are on the chromosome. The nomenclature used as an example OmpL36 WT+D is confusing as that implies if the WT allele is present and a second mutant allele is introduced. Authors can just refer to the modifications with subscript.

Response: Whilst this was briefly mentioned in the original text, we have expanded on it ensure that it is clear that all changes were made on the chromosome (lines 150-152). The nomenclature was established in previous work and all structural databases containing data on these proteins e.g. pdb. Whilst we appreciate that the reviewer found this confusing, we feel that modifying it now will make following work in the field difficult, especially for structural biologists who use this nomenclature routinely.

Comment 3: Also, the mutant alleles are initially mentioned in line 139, but only explained in 160-162. The authors can explain this when they first introduce the alleles.

Response: We are sorry for the confusion; the first mention refers to recombinant OmpK36 expressed in E. coli and the latter to OmpK36 expressed in KP; this has been mentioned for clarity (line 242).

Comment 4: Is OmpK35 deletion required for resistance? Do you require deletion/modification of both alleles? This is not addressed.

Response: We have addressed this comment the OmpK35 section of the introduction (lines 62-64). Mutations in OmpK35 appear to have little to no effect on modifying the MIC alone but do enhance the effect of OmpK36 resistance mutations. This has been demonstrated in two previous pieces of work which has been cited.
